
JOURNAL INFORMATION
==============================
NLM Title Abbreviation: ScientificWorldJournal
Journal ID: ScientificWorldJournal
NLM Journal ID: 101131163
Journal ID: TSWJ

The Scientific World Journal

ISSN: 2356-6140
EISSN: 1537-744X
Publisher: Wiley

ARTICLE INFORMATION
==============================
PMCID: PMC6084134
PMID: 30147589
DOI: 10.1100/tsw.2001.132
Article ID: 842193
Article version: 1
Subjects: Short Report

Hibernation, a Model of Neuroprotection

Zhou Fang 1 *
Smith Mark A. 1
Castellani Rudy J. 2
Stimmelmayr Raphaela 1
Perry George 2
Drew Kelly L. 1
Zhu Xiongwei 1
1 Institute of Arctic Biology Department of Chemistry and Biochemistry University of Alaska Fairbanks, Fairbanks, AK, USA
2 Institute of Pathology Case Western Reserve University, Cleveland, OH, USA
*Fang Zhou: ftfz@uaf.edu
Publication date: 2001
Electronic publication date: 2001 Dec 19
Volume: 1
First page: 108
Last page: 108
Copyright: Copyright © 2001 Fang Zhou et al.
Copyright year: 2001
License: This is an open access article distributed under the Creative Commons Attribution License, which permits unrestricted use, distribution, and reproduction in any medium, provided the original work is properly cited.
License URL: https://creativecommons.org/licenses/by/3.0/

Funding: American Heart Association 98-AK-301 National Institutes of Health NS38648 National Institutes of Health NS41069-01

==============================
Miami *Nature Biotechnology* Short Reports
*TheScientificWorld* (2001) 1(S3), 108SR
ISSN 1532-2246; DOI 10.1100/TSW.2001.132

### HIBERNATION, A MODEL OF NEUROPROTECTION

Fang Zhou*, Xiongwei Zhu[†], Mark A. Smith[†], Rudy J. Castellani[†], Raphaela Stimmelmayr, George Perry[†], and Kelly L. Drew

Institute of Arctic Biology and Department of Chemistry and Biochemistry, University of Alaska Fairbanks, Box 757000, Fairbanks, AK 99775 and [†]Institute of Pathology, CaseWestern Reserve University, 2085 Adelbert Rd. Cleveland, OH 44106
* ftfz@uaf.edu

**INTRODUCTION.** Hibernation, a natural model of tolerance to "cerebral ischemia", represents a state of pronounced fluctuation in cerebral blood flow where no brain damage occurs (1). Numerous neuroprotective aspects may contribute in concert to such tolerance. The purpose of this study was to determine if hibernating brain tissue is tolerant to penetrating brain injury modeled by insertion of microdialysis probes.

**METHODS.** Guide cannulae were surgically implanted in striatum of arctic ground squirrels before any of the animals began to hibernate. Microdialysis probes were then inserted in some animals after they entered hibernation and in others while they remained euthermic. After 3 days implantation of microdialysis probes, the brain tissue from hibernating and euthermic animals was examined by hematoxylin and eosin (H & E) stain and immunocytochemical identification of activated microglia, astrocytes and HO-1 immunoreactivity. Total white blood cell counts were performed on both hibernating and euthermic animals to assess leukocytopenia.

**RESULTS.** White blood cell counts decrease from $6357 \pm 2739/mm^3$ in euthermic animals to $456 \pm 98/mm^3$ in hibernating animals (mean ± SD, n=8-10, $p<0.0001$ main effect of state). Examination of H&E stained tissue sections indicated obvious histological changes, including reactive astrocytosis, microglial activation, macrophage infiltrate, and axonal swellings around the probe track in euthermic animals while there was no significant cellular tissue response around the probe track in hibernating tissue.

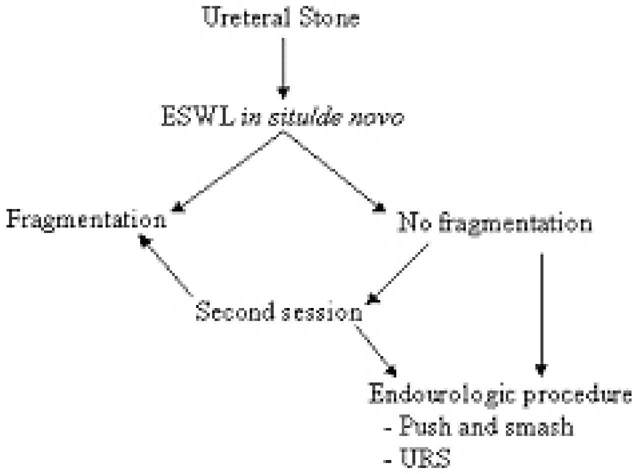

**Fig. 1.** Activated microglia identified by RCA-1 stain. Scale bar = 100 μm.

Activated microglia (Fig. 1) and astrocytes, identified by RCA-1 binding and GFAP immunoreactivity, respectively, was more intense around the probe tracts (arrow) in euthermic animals compared to hibernating animals. Hemeoxygenase-1 (HO-1) immunoreactivity, a marker of oxidative stress response induction, was dramatically attenuated around probe tracks in hibernating animals compared to euthermic controls.

**DISCUSSION.** The present results revealed by histopathological analysis suggest that hibernation is neuroprotective. In euthermic brain tissue the typical inflammatory response was evidenced by the presence of activated microglia and astrocytes and the oxidative stress response. However, these responses were profoundly suppressed in hibernating animals. The significant degree of protection during hibernation likely results from a combination of neuroprotective aspects, including hypothermia, leukocytopenia and an increase in extracellular concentrations of ascorbate during hibernation.

**ACKNOWLEDGEMENT.** This work was supported by the American Heart Association (AHA grant 98-AK-301), a UAF PSPF grant to K.L.D. The National Institutes of Health (grant NS38648 to M.A.S) and NS41069-01 SNRP funded in part by NINDS, NIMH and NCRR.

**REFERENCES.**

1. Lyman, C.P. (1948) J. Exp. Zool. 109, 55-78
2. Snapp, B.D. and Heller, C. (1981) Physiol. Zool. 54, 297-307
3. Frerichs, K.U., Smith, C.B., Brenner, M., et al. (1998) Proc. Natl. Acad. Sci. U S A 95(24), 14511-14516
4. Drew, K.L., Osborne, P.G., Frerichs, K.U., et al. (1999) Brain Res. 851(1-2), 1-8
